# Application of Mitochondrial and Oxidative Stress Biomarkers in the Evaluation of Neurocognitive Prognosis Following Acute Carbon Monoxide Poisoning

**DOI:** 10.3390/metabo12030201

**Published:** 2022-02-24

**Authors:** Yong Sung Cha, Jae Seung Chang, Hyun Kim, Kyu-Sang Park

**Affiliations:** 1 Department of Emergency Medicine, Wonju College of Medicine, Yonsei University, Wonju 26426, Korea; emyscha@yonsei.ac.kr; 2 Research Institute of Hyperbaric Medicine and Science, Wonju College of Medicine, Yonsei University, Wonju 26426, Korea; 3 Mitohormesis Research Center, Wonju College of Medicine, Yonsei University, Wonju 26426, Korea; godbless@yonsei.ac.kr; 4 Department of Physiology, Wonju College of Medicine, Yonsei University, Wonju 26426, Korea

**Keywords:** biomarkers, carbon monoxide poisoning, hyperbaric oxygen, growth differentiation factor 15, 8-Oxo-2′-deoxyguanosine

## Abstract

Mitochondrial and oxidative stress play critical roles in the pathogenic mechanisms of carbon monoxide (CO)-induced toxicity. This study was designed to evaluate whether the serum levels of specific stress biomarkers might reflect brain injury and act as prognostic markers for the development of neurocognitive sequelae following CO poisoning. We analyzed the data from 51 adult patients admitted with acute CO poisoning and measured the serum level expression of growth differentiation factor 15 (GDF15) and fibroblast growth factor 21 (FGF21), indicators of mitochondrial stress, and 8-Oxo-2′-deoxyguanosine (8-OHdG) and malondialdehyde (MDA), indicators of oxidative stress. Serum was collected upon arrival at the hospital, at 24 h post treatment, and within 7 days of HBO_2_ therapy. Global Deterioration Scale scores were measured 1 month post incident and used to place the patients in either favorable or poor outcome groups. Initial serum GDF15 and 8-OHdG concentrations were significantly increased in the poor-outcome group and all four biomarkers decreased at 24 h post HBO_2_ therapy, and were then maintained or further decreased at the 1-week mark. Notably, the degree of change in these biomarkers between baseline and 24 h post HBO_2_ were significantly larger in the poor-outcome group, reflecting greater CO-associated stress, confirming that post-CO poisoning serum biomarker levels and their response to HBO_2_ were proportional to the initial stress. We suggest that these biomarkers accurately reflect neuronal toxicity in response to CO poisoning, which is consistent with their activity in other pathologies.

## 1. Introduction

Carbon monoxide (CO) is a colorless, tasteless, odorless gas usually produced by the incomplete combustion of carbon compounds and common sources include fires, engine exhausts and faulty furnaces [1]. Each year, about 50,000 people are admitted to the emergency department (ED) for CO poisoning [2], which causes 1500 deaths in the USA every year [3]. Randomized control trials have shown that hyperbaric oxygen (HBO_2_) therapy is beneficial for symptomatic patients when administered within 24 h of CO poisoning [4]. Acute CO poisoning can produce serious neurologic sequelae, affecting the patient’s long-term prognosis. However, there are no known serum biomarkers for predicting post-CO poisoning prognosis.

One of the central pathophysiological mechanisms of CO toxicity is mediated via the inhibition of mitochondrial respiration. CO blocks electron-transport chain activity by binding to the ferrous hem within the cytochrome c oxidase (COX; complex IV) active site. This shuts down oxidative phosphorylation, similar to the effects of both cyanide and nitric oxide [1,5,6,7]. Since COX has a three-fold higher binding affinity for CO than O_2_, CO-mediated mitochondrial inhibition is significantly worse under hypoxic conditions due to a reduction in the competitive binding of O_2_ and CO [8,9]. COX inhibition induces a reduction in oxidative phosphorylation causing a decrease in adenosine triphosphate production within the affected tissues [1,10]. However, because other complexes in the electron transport chain continue to shuttle electrons, there is an increase in superoxide production leading to further injury of cells and tissues [11]. Oxidative stress induced by CO poisoning is also implicated in the serious neurologic complications associated with CO exposure. Acute CO poisoning causes neutrophil degranulation [12] and induces the release of myeloperoxidase (MPO), proteases, and reactive oxygen species [12]. This oxidative stress leads to xanthine dehydrogenase transformation to xanthine oxidase in the endothelial cells, as well as lipid peroxidation and apoptosis [12,13].

Growth differentiation factor 15 (GDF15) is known to regulate food intake and energy metabolism while fibroblast growth factor 21 (FGF21) is also known to be involved in nutrient metabolism and adaptive thermogenesis. Interestingly, increased GDF15 and FGF21 expression has been shown to exert some protective effects against various pathogenic conditions including heart failure and obesity [14]. Recent studies have also shown that both GDF15 and FGF21 are induced by mitochondrial stress and are commonly referred to as stress hormones [15]. Circulating GDF15 and FGF21 levels can be used to predict the diagnosis and prognosis of metabolic syndrome and cardiovascular diseases [16,17], while 8-Oxo-2′-deoxyguanosine (8-OHdG) and malondialdehyde (MDA) have been frequently used as serum biomarkers for oxidative stress in cancer, psychiatry, chronic obstructive pulmonary disease, asthma, and various cardiovascular diseases [18,19].

We previously reported that HBO_2_ therapy significantly reduced mitochondrial stress biomarker expression in a human volunteer study, suggesting a potential beneficial effect for the application of HBO_2_ in mitigating mitochondrial stress [20]. Given their clear application in other pathologic conditions, we hypothesized that the severity of the neurocognitive sequelae induced following acute CO poisoning may be correlated with serum mitochondrial (GDF15 and FGF21) and oxidative stress (8-OHdG and MDA) biomarkers. Therefore, we designed this study to investigate whether the severity of neurocognitive sequelae following acute CO poisoning correlates with serum mitochondrial and oxidative stress biomarkers and if HBO_2_ therapy might reduce these effects.

## 2. Results

### 2.1. Characteristics of the Study Population

Only 51 of the 153 patients with CO poisoning who visited the ED between January 2020 and January 2021 were included in this study (Supplementary Materials Figure S1), 38 (74.5%) of whom were included in the favorable neurocognitive outcome group and 13 (25.5%) of whom experienced a poor neurocognitive outcome. Table 1 summarizes the demographic and baseline characteristics of the patient cohort and we noted that the poor outcome group were largely older (*p* < 0.001) and had a longer CO exposure time (*p* = 0.002) than the favorable outcome group. The mean GCS score was significantly lower (*p* = 0.006), and shock occurred more frequently (*p* = 0.046) in the poor outcome group. In addition, all of the evaluated complications were significantly more severe in the poor outcome group, with serum lactate (*p* = 0.042), creatine kinase (*p* < 0.001), and troponin I (*p* < 0.001) levels all increasing in the poor outcome group when compared to the favorable outcome group. Bicarbonate levels (*p* = 0.005) were also lower in the poor outcome group.

### 2.2. Changes in Stress Biomarkers Following Hyperbaric Oxygen Therapy

**Figure 1:**
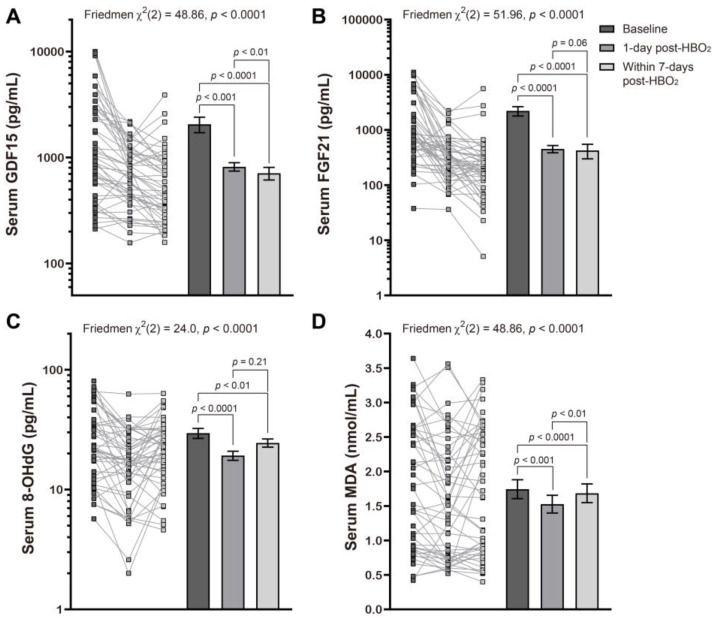
Effects of hyperbaric oxygen treatment on the serum levels of several mitochondrial and oxidative stress biomarkers. Changes in serum levels of GDF15 (**A**), FGF21 (**B**), 8-OHdG (**C**), and MDA (**D**) at baseline, l day post and within 7 days of HBO_2_ therapy, in patients with CO poisoning were evaluated using a Friedman test followed by a Dunn’s multiple comparison correction. Symbols represent the individual values from the patient cohort while the bars represent the mean ± standard error of the mean for each group. Note that the *y* axis is described using the log scale in panels (**A**–**C**).

We found that the mean mitochondrial and oxidative stress indicator values were markedly decreased when compared with baseline and remained lower for up to 1-week following HBO_2_ therapy. This was most obvious with GDF15 which continued to decrease with time (Figure 1), all of which was consistent with our previous report [20]. 

**Figure 2:**
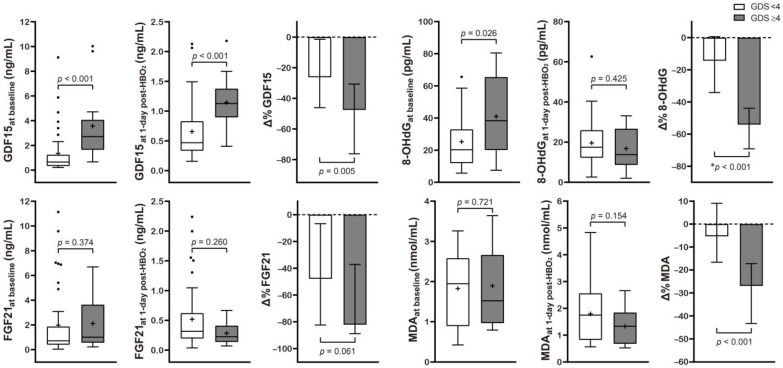
Serum level concentration of various mitochondrial and oxidative stress biomarkers in CO poisoning patients presenting with different neurocognitive outcomes. Data are presented as box and whisker Tukey plots with medians and interquartile ranges (+, mean; •, outliers). Comparisons of the biomarker levels between the favorable (GDS < 4) and poor (GDS ≥ 4) neurocognitive outcome groups were completed using a two-sided Mann–Whitney U test or * independent *t*-test, as appropriate. The change (Δ%) in each parameter was calculated as follows: (1 day post HBO_2_ therapy value − baseline value)/baseline value test) × 100.

Table 2 and Figure 2 present the correlation between neurocognitive sequelae and serum GDF15, FGF21, 8-OHdG, and MDA levels. Baseline GDF15 (*p* < 0.001) and 8-OHdG (*p* = 0.026) were significantly higher in the poor outcome group than in the favorable outcome group. HBO_2_ therapy induced significantly increased Δ% GDF15 (*p* = 0.005), FGF21 (*p* = 0.061), 8-OHdG (*p* < 0.001), and MDA (*p* < 0.001) in the poor outcome group.

### 2.3. Stratification of the Serum Mitochondrial and Oxidative Stress Biomarkers Based on Neurocognitive Outcome Following CO Poisoning

The AUROCs for Δ% of GDF15, 8-OHdG, and MDA were 0.757, 0.885, and 0.828, respectively (Supplementary Materials Figure S2). While univariate analyses revealed that the Δ% of both the oxidative and mitochondrial stress indicators (Wald χ^2^: 10.52 for Δ% 8-OHdG, *p* = 0.001; 9.27 for Δ% MDA, *p* = 0.002; 6.41 for Δ% GDF15, *p* = 0.011) were significantly associated with neurocognitive outcome, with these correlations being superior or comparable to that observed with CO exposure time (χ^2^ = 7.86, *p* = 0.005) and GCS at the ED (χ^2^ = 6.08, *p* = 0.014).

### 2.4. Potential Predictive Models for Neurocognitive Outcome Prognoses

**Figure 3:**
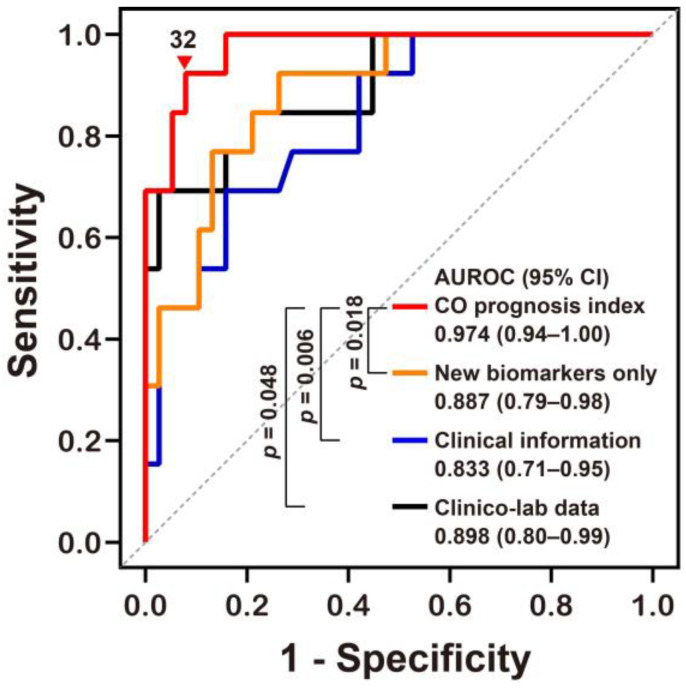
Receiver operating characteristic curve analyses of the novel prognostic index for predicting neurocognitive outcomes following CO poisoning. The equation for predicting CO poisoning-induced neurocognitive sequelae can be explained as follows: CO prognosis index = 1/(1 + exp[−(−0.081 × Δ% of GDF15 − 0.169 × Δ% of MDA + 0.368 × CO exposure time (h) − 0.688 × GCS at the emergency department − 3.221)] × 100. AUROC, area under the receiver operating characteristic curve; CI, confidence intervals; MDA, malondialdehyde; GDF15, growth differentiation factor 15; CO, Carbon monoxide; GCS, Glasgow Coma Scale. Other equations derived from the stress biomarkers alone (New biomarkers alone) and clinical information with or without (Clinical information) laboratory data (Clinico-lab data) were compared to our novel CO prognosis index and the optimal cut-off value (▼) for this new index was defined using the Youden index value. All *p* values were obtained using a DeLong’s test.

We derived a potential prediction model (called the “CO prognosis index”), which included the best performing stress biomarkers. We then used a multivariate stepwise logistic regression analysis, with adjustment for variables with *p* values of <0.05 on univariate analyses, and identified Δ% MDA, GCS at the ED, Δ% GDF15, and CO exposure time (h) as significant independent predictors (rank-ordered by Wald χ^2^) for neurocognitive impairment and included these in our index (Table 3). Our CO prognosis index had excellent predictive capability with an AUROC value of 0.974 (Figure 3 and Supplementary Materials Table S2). The diagnostic power of the CO prognosis index was significantly higher than that of other models comprising the novel stress biomarkers alone, clinical information alone, or clinical information with routine laboratory data (Table 3 and Figure 3).

## 3. Discussion

Mitochondrial and oxidative stresses are important pathogenic molecular mechanisms involved in the development of neurocognitive complications in patients with acute CO poisoning. This led us to assume that the evaluation of these cellular stresses using serum biomarkers might help predict pathological progression, severity, and prognosis related to serious complications. In this study, we demonstrated that serum biomarkers reflecting mitochondrial and oxidative stresses demonstrate a significant correlation with neurocognitive outcomes following CO poisoning, which have not been reported previously. Interestingly, we noted that CO poisoning patients with poor neurocognitive outcomes demonstrated more significant changes in their mitochondrial and oxidative stress biomarker response to HBO_2_ therapy. These results imply that these patients experienced a greater decrease in these biomarker levels following HBO_2_ therapy which may suggest more serious initial damage following CO-related mitochondrial and oxidative stress. Therefore, changes in these stress biomarkers after HBO_2_ therapy could be a useful indicator of the degree of CO toxicity in patients entering hospital EDs.

Functional impairment of the mitochondria accompanied by increased superoxide production is also associated with metabolic syndrome and age-related morbidities. Vicious cycles involving oxidative, mitochondrial, and endoplasmic reticulum stress are critically associated with the pathogenic mechanisms of type 2 diabetes and metabolic syndrome [21]. To overcome these cellular stresses and to abate the pathological progression of metabolic diseases, the body induces the integrated stress response (ISR) to enable metabolic and mitochondrial flexibility. The ISR includes secretion of mitochondrial stress-inducible humoral factors, such as GDF15 and FGF21 [22]. However, sustained and uncompensated mitochondrial stress maintains these elevated GDF15 and FGF21 levels which may have other pathological effects [14].

GDF15 and FGF21 are expressed mainly in the liver, fat, muscle, kidney, lung, and pancreas, and are induced by activating transcription factor 4 (ATF4) from the ISR [23,24,25,26]. Various stresses and tissue injuries upregulate GDF15 and FGF21 expression and result in their greater release into the circulation, allowing them to suppress inflammation and prevent disease progression. GDF15 is also expressed in the choroid plexus, where it acts as a potent neurotrophic factor for motor and sensory neurons [23]. The role of serum GDF15 has been evaluated in large patient cohorts as a biomarker for mitochondrial dysfunction, obesity, diabetes, cardiovascular diseases, ageing, and age-related disorders [17,23,24]. FGF21 also plays a protective role against metabolic and oxidative stresses, as demonstrated by its upregulation in critical illnesses and its role in inducing antioxidant protein expression [27,28].

Here, circulating concentrations of both GDF15 and FGF21 were elevated in response to acute CO poisoning and notably, serum GDF15 was shown to exert a better discriminatory effect for poor neurocognitive outcomes following CO intoxication than FGF21. This finding was consistent with a previous report which revealed that GDF15 had a higher sensitivity and specificity than FGF21 when used as a biomarker for mitochondrial diseases associated with respiratory chain defects [24]. Furthermore, FGF21 plays a negligible role in the metabolism during mitochondrial stress adaptation in the muscles [29], and both GDF15 and FGF21 have been reported to play significantly different roles in the systemic adaptation to mitochondrial ISR [30]. Each stress marker may display its own pattern of changes based on disease conditions; therefore, simultaneous measurement of GDF15 and FGF21 often improve disease-detection [14,23].

The amelioration of brain injury by HBO_2_ therapy occurs via improved oxidative phosphorylation [7], suppressed oxidative stress and cytokine release [31], inhibited lipid peroxidation [32], impaired leukocyte adhesion to injured microvasculature [33], and reduced neuronal inflammation caused by CO-induced adduct formation in the myelin basic protein [34]. Experimental evidence has shown that HBO_2_ therapy protects against mitochondrial dysfunction [35] and improves mitochondrial biogenesis and respiration [36,37], thus supporting its beneficial and therapeutic effects. Furthermore, HBO_2_ therapy can reverse CO binding of COX [7,38] and reduce oxidative stress via various mechanisms, including increased hem oxygenase-1 [39,40], the upregulation of antioxidant enzymes [31,41], and the induction of the heat shock protein levels, which protects against oxidative stress-induced damage [42].

The initial mean GDF15 and FGF21 values of patients with CO poisoning were higher in this study than in our previous study on healthy adult volunteers (GDF15: 1.92 vs. 0.73 ng/mL; FGF21: 2.02 vs. 0.42 ng/mL) [20]. In addition, indirect comparisons of the 8-OHdG and MDA values from the patients in this cohort and that of other studies [43,44] revealed an increase in 8-OHdG and MDA in these patients (8-OHdG: 29.2 vs. 25.5 pg/mL; MDA: 2.02 vs. 1.25 nmol/mL). Elevated serum biomarkers in the early stages of CO poisoning demonstrate that acute CO exposure induces mitochondrial and oxidative stress. These comparisons agree with our finding that the poor neurocognitive outcome group present with increased initial stress biomarkers.

This study also established a possible predictive model using regression analysis and stress indicators to predict neurological sequelae following CO poisoning. This novel index showed superior performance for the prediction of neurocognitive prognosis by measuring serological markers of mitochondrial and oxidative stress than either the biomarkers alone or the clinical evaluations alone could produce. These values can be easily obtained via simple peripheral blood tests and can be combined with the patient’s medical history and physical examination to produce a clear matrix for prognosis. The addition of stress biomarkers significantly improves prognosis predictability compared with other models, which comprise clinical information, with or without routine laboratory results. This model, and our study in general, act as a proof of concept for the application of mitochondrial and oxidative stress markers as critical indicators for the pathophysiological impacts of CO-toxicity. Our data also show that these effects can be attenuated by the timeous application of HBO_2_ therapy. We suggest that the larger the reduction in stress biomarker expression following HBO_2_ therapy the more severe the negative impact of CO poisoning in relation to neurocognitive function and thus a poorer prognosis. However, given the high variability in the serum level expression of these biomarkers between individuals, the decrement change (Δ%) following HBO_2_ therapy is likely to be a better indicator for prognosis.

Despite the promising results of our evaluation, this study did have a few limitations. First, only a single tertiary medical center was involved in this study. In addition, since this study was conducted on individuals in Korea alone, the inference drawn based on this study population may or may not be applicable to other populations. Further studies on other populations are needed. Nonetheless, this is the first study of its kind, and we hope that more will follow. Second, due to the different lengths of hospitalization for each patient, the same serial follow-up was not performed for all patients. Third, oxidative stress increases with age. Given this, we note that while the median age was significantly higher in the “poor outcome” group, our multivariate logistic regression analysis suggests that age was not a discriminating variable for neurocognitive outcomes and thus was not used as an independent factor in our novel prognostic model. However, there may be some bias as a result of these age-related effects in the oxidative stress data which should be examined in the future. Fourth, evaluation of the pyruvate levels in specific samples might increase the reliability of our mitochondrial evaluations by facilitating an examination of the lactate/pyruvate ratio. However, we did not measure the pyruvate level in this study and will consider this in future evaluations. Prognostic prediction models need to be validated in future large-scale studies. However, our data suggest that future large multicenter studies with a multi-modal prognostic scheme are warranted to improve the usefulness and effectiveness of this approach. 

## 4. Materials and Methods

### 4.1. Study Design and Setting

This was a prospective observational study designed to evaluate consecutive patients requiring HBO_2_ therapy for acute CO poisoning, admitted to the ED at Wonju Severance Christian Hospital (Wonju, Korea), between January 2020 and January 2021. The ED of this suburban, tertiary-care academic hospital records more than 46,000 visits annually and is staffed 24 h/day by board-certified emergency physicians. This study was approved by our hospital’s institutional review board (approval number: CR319100), and the study protocol complied with the ethical guidelines set out in the Declaration of Helsinki. Written informed consent was obtained from all participants and we anonymized the patient data before performing the analyses. This study has been registered at ClinicalTrials.gov, accessed on 1 December 2021 (NCT05088005).

The exclusion criteria are as follows: (1) age < 19 years; (2) a history of inherent mitochondrial disorders that might affect the primary outcome biomarkers [45]; (3) co-ingestion of drugs and alcohol, which may affect these biomarkers; (4) discharge from the ED or transfer to another hospital; (5) refusal to undergo HBO_2_ therapy; (6) refusal to enroll in this study; and (7) a lack of follow-up and neurocognitive assessment.

The diagnosis of CO poisoning was made based on the patient’s medical history and carboxyhemoglobin (CO-Hb) level > 5% (>10% in heavy smokers). Co-ingestion of drugs was confirmed using blood and urine screening tests, performed by the Forensic Toxicology Division at the National Forensic Service (Wonju, Korea). Serum ethanol levels were also evaluated to confirm alcohol ingestion. 

We treated patients with CO poisoning using 100% O_2_ therapy supplied through a face mask with a reservoir bag. Patients who had an interval of loss of consciousness, neuropsychologic symptoms and signs, cognitive dysfunction, cardiovascular dysfunction, severe acidosis, or CO-Hb ≥25% were treated using HBO_2_ administered via a multi-place or mono-place hyperbaric chamber [46]. HBO_2_ therapy sessions included an initial compression up to 2.8 atmospheres absolute for 45 min, followed by 2 atmospheres absolute for 60 min.

### 4.2. Biomarkers of Mitochondrial and Oxidative Stress

Blood samples were collected from the antecubital vein and placed in serum separation tubes which were then centrifuged at 3000 rpm (1000 g-force) for 10 min before the serum was collected and immediately stored at −80 °C for further analysis. Serum biomarkers of mitochondrial, GDF15 and FGF21, and oxidative stress, 8-OHdG and MDA, were measured upon arrival at the ED (0 h) and at 24 h post and within 7 days of HBO_2_ therapy. In the case of discharge before 7 days, measurements were taken at the time of discharge. We also investigated the percentage change (Δ%) in these variables which was defined as (((value at 24-h post-HBO_2_ therapy completion − baseline value)/baseline value)× 100) for each of the four stress markers when comparing baseline and 24-h post-HBO_2_ therapy values.

Serum GDF15 and FGF21 concentrations were quantified using commercial enzyme-linked immunosorbent assay kits (DGD150, DF2100; R&D System, Minneapolis, MN, USA). Serum 8-OHdG levels were analyzed by liquid chromatography-tandem mass spectrometry (API-4000, Applied Biosystems, Foster City, CA, USA), after sample purification by solid-phase extraction. 8-OHdG (Sigma–Aldrich, St Louis, MO, USA) and ^15^N_5_ 8-OHdG (Cambridge Isotope Laboratories, Andover, MA, USA) were used as the reference and internal standard, respectively. MDA levels were measured as MDA-thiobarbituric acid complex via high-performance liquid chromatography (Agilent 1200 HPLC series, Agilent, Santa Clara, CA, USA) using 1,1,3,3-tetramethoxypropan (Sigma–Aldrich, St. Louis, MO, USA) as a standard. Fluorometric detection was performed using 527-nm excitation and 551-nm emission.

### 4.3. Study Variables and Definitions

The following clinical variables were evaluated: age, sex, intention (suicide or accident), CO source (non-fire or fire), maximal CO exposure time (h), time from rescue to ED arrival (h), Glasgow Coma Scale (GCS) score at ED upon arrival, current smoking and drinking status, medical and psychiatric comorbidities, any interval of loss of consciousness, shock and seizure, and duration from the time of rescue to HBO_2_ administration (h). Maximal CO exposure time was defined as the estimated maximum duration from the time of the most recent episode of normal consciousness to the time of rescue. Complications (rhabdomyolysis, acute kidney injury, myocardial injury [defined as troponin I > 45.43 pg/mL], and pneumonia) during hospital admission were also investigated, and recorded using the following laboratory variables: CO-Hb blood levels, bicarbonate, and lactate levels in the arterial blood gas analysis, creatinine, creatine kinase, and troponin I at ED arrival.

We evaluated neurocognitive outcomes using the Global Deterioration Scale (GDS), a validated scoring system (range: 1–7) used for dementia assessment (Supplementary Materials Method S1 and Table S1) [47]. A higher GDS score indicates greater severity. The patients visited the rehabilitation outpatient department for their GDS evaluation 1 month post CO exposure. If the patient was unable to visit the outpatient department due to worsening condition, the patient’s guardians were interviewed. We classified the outcomes in terms of GDS scores as favorable (1–3 points) or poor (4–7 points) [48,49].

### 4.4. Study Outcomes

We used serum mitochondrial (GDF15 and FGF21) and oxidative stress (8-OHdG and MDA) biomarker levels pre- and post-HBO_2_ therapy as primary indicators in this study, comparing these values to assess whether HBO_2_ reduced stress. The secondary outcome of this study was the evaluation of these biomarkers as prognostic tools for neurocognitive outcomes 1 month post trauma.

### 4.5. Statistical Analyses

Data for continuous variables were presented as the median (interquartile range) or mean ± standard deviation while categorical variables were recorded as frequencies (percentages). Continuous data were tested for normality using the Shapiro–Wilk test and subgroups were compared using independent *t*-test or Mann–Whitney U test for continuous variables and the chi-square or Fisher’s exact tests for categorical variables. We used pairwise repeated-measure analysis of variance to assess individual changes in serum biomarker levels between the baseline and follow-up samples. These evaluations were completed using a Friedman test and Dunn’s multiple comparison correction. Variables with a *p* < 0.1 in the univariate analyses were then evaluated in the multivariate analysis using stepwise selection. The prognostic powers of prediction models were evaluated using area under the receiver operator characteristic curve (AUROC) analyses, with assessments of likelihood ratios, predictive values, and accuracy. The optimal cut-off values were determined using the Youden index and all statistical analyses were performed using SPSS 25.0 software (IBM Corp., Armonk, NY, USA) and Prism 8.0 (GraphPad Software, Inc., San Diego, CA, USA). Two-sided *p* values < 0.05 were considered statistically significant.

## 5. Conclusions

This study suggests that serum biomarkers for mitochondrial and oxidative stress correlate with the neurocognitive outcomes of CO poisoning. The changes in these biomarkers following HBO_2_ therapy are proportional to the initial stress imposed by CO poisoning and we suggest that serial measurements of these biomarkers could provide more information around the pathological stress burdens and disease progression of neurocognitive disorders and facilitate improved prognostic evaluations in not only acute CO toxicity but also other chronic metabolic and neurodegenerative diseases.

## Figures and Tables

**Table 1:** Demographic and baseline characteristics of the patient cohort.

	Total	Favorable Outcome	Poor Outcome	*p*-Value
N	51 (100)	38 (74.5)	13 (25.5)	
Age (years)	47 (32, 60)	41 (24, 49)	60 (52, 71)	<0.001
Sex (male)	41 (80.3)	30 (78.9)	11 (84.6)	0.999
Intention of self-harm	25 (49)	21 (55.3)	4 (30.8)	0.199
Source of CO				
No fire	48 (94.1)	35 (92.1)	13 (100)	0.561
Fire	3 (5.9)	3 (7.9)	0 (0)	
Maximal CO exposure time (h)	3.5 (1.48, 8)	2.71 (1, 5)	8 (4.92, 12)	0.002
Time from rescue to ED (h)	3.07 (1.52, 4.85)	2.98 (1.73, 4.6)	3.35 (1.48, 8.3)	0.482
GCS at the ED	12 (8, 15)	12 (8, 15)	8 (8, 12)	0.006
Current smoker	22 (43.1)	19 (50)	3 (23.1)	0.114
Current drinker *	22 (43.1)	17 (44.7)	5 (38.5)	0.693
Comorbidities				
Diabetes mellitus	7 (13.7)	4 (10.5)	3 (23.1)	0.352
Hypertension	8 (15.7)	4 (10.5)	4 (30.8)	0.179
Dyslipidaemia	7 (13.7)	5 (13.2)	2 (15.4)	0.999
Lung diseases	1 (2)	1 (2.6)	0 (0)	0.999
Cardiovascular diseases	3 (5.9)	3 (7.9)	0 (0)	0.561
Liver diseases	1 (2)	1 (2.6)	0 (0)	0.999
Psychiatric diseases	7 (13.7)	7 (18.4)	0 (0)	0.169
Symptoms and signs at the ED				
Loss of consciousness	43 (84.3)	30 (78.9)	13 (100)	0.096
Shock	4 (7.8)	1 (2.6)	3 (23.1)	0.046
Seizure	1 (2)	1 (2.6)	0 (0)	0.999
Time elapsed from rescue to HBO_2_ therapy (h)	4.97 (3.37, 9.08)	4.63 (3.3, 7.09)	6.58 (4.17, 16.1)	0.098
Biochemical markers				
CO-Hb (%) ^#^	24.1 ± 15.2	23.1 ± 15.8	27.2 ± 13.5	0.294
Bicarbonate (mmol/L) ^#^	22 ± 4.1	23 ± 3	18.8 ± 5.2	0.005
Lactate (mmol/L)	2.3 (1.4, 3.3)	2.2 (1.2, 3.1)	2.7 (2.1, 8.5)	0.042
Creatine kinase (U/L)	143 (103, 370)	129 (95, 212)	1379 (299, 7531)	<0.001
Troponin I (pg/mL)	38.8 (4.2, 541.5)	12.1 (2.5, 229.6)	541.5 (282, 2884)	<0.001
Complications				
Acute kidney injury	4 (7.8)	0 (0)	4 (30.8)	0.003
Rhabdomyolysis	12 (23.5)	2 (5.3)	10 (76.9)	<0.001
Myocardial injury	25 (49.0)	13 (34.2)	12 (92.3)	<0.001
Pneumonia	10 (19.6)	2 (5.3)	8 (61.5)	<0.001

Data are presented as the median (interquartile range), mean ± standard deviation, or *n* (%). Categorical variables were compared using Fisher exact test or * chi-square test. Continuous variables were compared with the Mann–Whitney U test or ^#^ independent *t*-test. CO, carbon monoxide; ED, emergency department; GCS, Glasgow Coma Scale; HBO_2_, hyperbaric oxygen therapy; CO-Hb, carboxyhemoglobin.

**Table 2:** Summary of the biomarkers values from the patient cohort.

	Total	Favorable Outcome	Poor Outcome	*p*-Value
GDF15_initial_ (ng/mL)	0.92 (0.4, 2.72)	0.65 (0.33, 1.25)	2.72 (1.65, 4.07)	<0.001
FGF21_initial_ (ng/mL)	0.8 (0.44, 1.85)	0.71 (0.4, 1.87)	1.01 (0.56, 3.63)	0.374
8OHdG_initial_ (pg/mL)	22.1 (12.9, 38.3)	20.3 (12.0, 33.0)	38.3 (20.1, 65.5)	0.026
MDA_initial_ (nmol/mL)	1.94 (0.9, 2.61)	1.95 (0.89, 2.58)	1.52 (0.97, 2.66)	0.721
GDF15_1d-HBO2_ (ng/mL)	0.66 (0.37, 1.13)	0.47 (0.33, 0.83)	1.13 (0.89, 1.37)	<0.001
FGF21_1d-HBO2_ (ng/mL)	0.31 (0.17, 0.54)	0.32 (0.19, 0.62)	0.22 (0.14, 0.41)	0.260
8-OHdG_1d-HBO2_ (pg/mL)	17.3 (11.7, 25.5)	17.5 (12.2, 26.0)	13.8 (8.7, 26.7)	0.425
MDA_1d-HBO2_ (nmol/mL)	1.56 (0.76, 2.36)	1.75 (0.82, 2.56)	1.33 (0.68, 1.84)	0.154
Δ% of GDF15	−33.4 (−55.9, −11.7)	−26.2 (−46.1, −1.4)	−47.6 (−76.3, −30.7)	0.005
Δ% of FGF21	−63.0 (−84.9, −10.0)	−47.8 (−82.4, −6.7)	−82.1 (−88.9, −37.2)	0.061
Δ% of 8-OHdG ^#^	−25.0 ± 33.6	−14.4 ± 31.0	−56.1 ± 18.0	<0.001
Δ% of MDA	−9.2 (−23.2, 5.3)	−5.3 (−16.6, 9.0)	−26.9 (−43.3, −17.3)	<0.001

Data are presented as the median (interquartile range) or mean ± standard deviation. *p*-values were obtained using the Mann–Whitney U test or ^#^ independent *t*-test, as appropriate. Initial, serum biomarkers were measured at the time of arrival at the Emergency Department (0 h); 1d-HBO_2_, serum biomarkers were evaluated 24 h post HBO_2_ therapy; GDF15, growth differentiation factor 15; FGF21, fibroblast growth factor 21; 8-OHdG, 8-Oxo-2′-deoxyguanosine; MDA, malondialdehyde. The change (Δ%) in each parameter was calculated as follows: (1 day post HBO_2_ therapy value − initial value)/initial value test) × 100.

**Table 3:** Logistic regression analyses for predicting the neurocognitive outcome of post-CO poisoning.

Variables	Univariate Analyses	Multivariate Analyses (Adjusted OR)			
	Unadjusted OR	CO Prognosis Index	New Biomarkers Alone	Clinical Information	Clinico-Lab Data
Age (years)	1.07 (1.02–1.12)	–	N/A	–	–
GCS at the ED	0.76 (0.61–0.94)	0.50 (0.27–0.94)	N/A	0.76 (0.60–0.97)	0.74 (0.56–0.99)
CO exposure time (h)	1.22 (1.06–1.41)	1.45 (1.01–2.06)	N/A	1.21 (1.05–1.40)	1.19 (1.01–1.40)
Time elapsed from rescue to HBO_2_ (h)	1.15 (1.02–1.29)	–	N/A	–	–
Bicarbonate (mmol/L)	0.76 (0.63–0.93)	–	N/A	N/A	–
Lactate (mmol/L)	1.38 (1.04–1.83)	–	N/A	N/A	–
Creatine kinase (mU/L)	2.22 (0.99–4.97)	–	N/A	N/A	2.54 (1.07–6.05)
Troponin I (µg/mL)	2.06 (1.03–4.12)	–	N/A	N/A	–
GDF15_baseline_ (ng/mL)	1.47 (1.07–2.01)	–	–	N/A	N/A
8OHdG_baseline_ (pg/mL)	1.04 (1.01–1.08)	–	–	N/A	N/A
GDF15_1d-HBO2_ (ng/mL)	6.17 (1.59–23.9)	–	–	N/A	N/A
MDA_1d-HBO2_ (nmol/mL)	0.55 (0.25–1.22)	–	–	N/A	N/A
Δ% of GDF15	0.97 (0.94–0.99)	0.92 (0.85–1.00)	0.96 (0.92–0.99)	N/A	N/A
Δ% of 8OHdG	0.94 (0.90–0.98)	–	–	N/A	N/A
Δ% of MDA	0.93 (0.89–0.98)	0.84 (0.74–0.96)	0.92(0.88–0.97)	N/A	N/A

Values are expressed as odds ratio (OR), with the 95% confidence interval in parentheses. GCS, Glasgow Coma Scale; ED, emergency department; CO, carbon monoxide; HBO_2_, hyperbaric oxygen therapy; GDF15, growth differentiation factor 15; FGF21, fibroblast growth factor 21; 8-OHdG, 8-Oxo-2′-deoxyguanosine; MDA, malondialdehyde; N/A, not applicable.

## Data Availability

All the data described in this study are available within the article or its Supplementary Materials.

## Supplementary Materials

The following are available online at https://www.mdpi.com/article/10.3390/metabo12030201/s1 [47,50,51,52,53]. Method S1: Description of the global deterioration scale. Figure S1: Flow diagram describing the study protocol. Figure S2: Receiver operating characteristic curve analyses of each of the stress biomarkers for predicting neurocognitive outcomes following CO poisoning. Table S1: Global deterioration scale values. Table S2: Predictive performance of the CO prognosis index in the evaluation of the neurocognitive outcomes following CO poisoning.
