# Supplementary material for: Application of Mitochondrial and Oxidative Stress Biomarkers in the Evaluation of Neurocognitive Prognosis Following Acute Carbon Monoxide Poisoning
Source: Metabolites. 2022 Feb 24;12(3):201. doi: 10.3390/metabo12030201 (PMC8952273; doi:10.3390/metabo12030201)
Supplement: Supplementary file 1 [file metabolites-12-00201-s001.zip › metabolites-1556122-supplementary.pdf]

Supplementary Information

Application of Mitochondrial and Oxidative Stress Biomarkers in the Evaluation of Neurocognitive Prognosis Following Acute Carbon Monoxide Poisoning

Yong Sung Cha [1,2,†], Jae Seung Chang [3,4,†], Hyun Kim [1,2,*] and Kyu-Sang Park [3,4,*]

[1] Department of Emergency Medicine, Wonju College of Medicine, Yonsei University, Wonju 26426, Korea; emyscha@yonsei.ac.kr
[2] Research Institute of Hyperbaric Medicine and Science, Wonju College of Medicine, Yonsei University, Wonju 26426, Korea
[3] Mitohormesis Research Center, Wonju College of Medicine, Yonsei University, Wonju 26426, Korea;
godbless@yonsei.ac.kr
[4] Department of Physiology, Wonju College of Medicine, Yonsei University, Wonju 26426, Korea
* Correspondence: khyun@yonsei.ac.kr (H.K.); qsang@yonsei.ac.kr (K.-S.P.); Tel.: +82-33-741-1615 (H.K.); Tel.: +82-33-741-0294 (K.-S.P.)
† These authors contributed equally to this work and share co-first authorship.

Supplementary Method S1. Global deterioration scale.

The Global Deterioration Scale (GDS) is a validated, reliable instrument for describing the clinical progression of dementia [1]. It is also used to determine the prognosis of patients with carbon monoxide (CO) poisoning [2]; and those with severe chronic obstructive pulmonary disease, Alzheimer's disease, and vasculopathy-related dementia [1,3-5].

Although the GDS score is not as diverse as a CO battery, it has the advantage of being able to identify neurocognitive functions, such as memory and concentration, as well as activities of daily living, through interviews. Moreover, many neurocognitive function tests may be difficult to administer in patients with sequelae. The Short-Form General Health Survey-36, a commonly used testing tool, has a set of self-reported questions; however, it is limited in evaluating patients with severe neurological impairment as it requires an individual's ability to understand and address the questions. Digit span, trail making, and clock drawing are good evaluation tools, but require short-term memory and visuospatial functions. Therefore, the GDS score can be used for all patients with CO poisoning, regardless of poisoning severity. The scale comprises seven scores, with higher scores indicating greater severity.

References

1. Reisberg, B.; Ferris, S.H.; de Leon, M.J.; Crook, T. The global deterioration scale for assessment of primary degenerative dementia. *Am J Psychiatry* **1982**, *139*, 1136-1139, doi:10.1176/ajp.139.9.1136.
2. Kim, S.J.; Thom, S.R.; Kim, H.; Hwang, S.O.; Lee, Y.; Park, E.J.; Lee, S.J.; Cha, Y.S. Effects of adjunctive therapeutic hypothermia combined with hyperbaric oxygen therapy in acute severe carbon monoxide poisoning. *Critical care medicine* **2020**, *48*, e706-e714, doi:10.1097/ccm.0000000000004419.
3. Paul, R.H.; Cohen, R.A.; Moser, D.J.; Zawacki, T.; Ott, B.R.; Gordon, N.; Stone, W. The global deterioration scale: Relationships to neuropsychological performance and activities of daily living in patients with vascular dementia. *J Geriatr Psychiatry Neurol* **2002**, *15*, 50-54, doi:10.1177/089198870201500110.
4. Eisdorfer, C.; Cohen, D.; Paveza, G.J.; Ashford, J.W.; Luchins, D.J.; Gorelick, P.B.; Hirschman, R.S.; Freels, S.A.; Levy, P.S.; Semla, T.P., *et al.* An empirical evaluation of the global deterioration scale for staging alzheimer's disease. *Am J Psychiatry* **1992**, *149*, 190-194, doi:10.1176/ajp.149.2.190.
5. Ozge, C.; Ozge, A.; Unal, O. Cognitive and functional deterioration in patients with severe copd. *Behav Neurol* **2006**, *17*, 121-130, doi:10.1155/2006/848607.

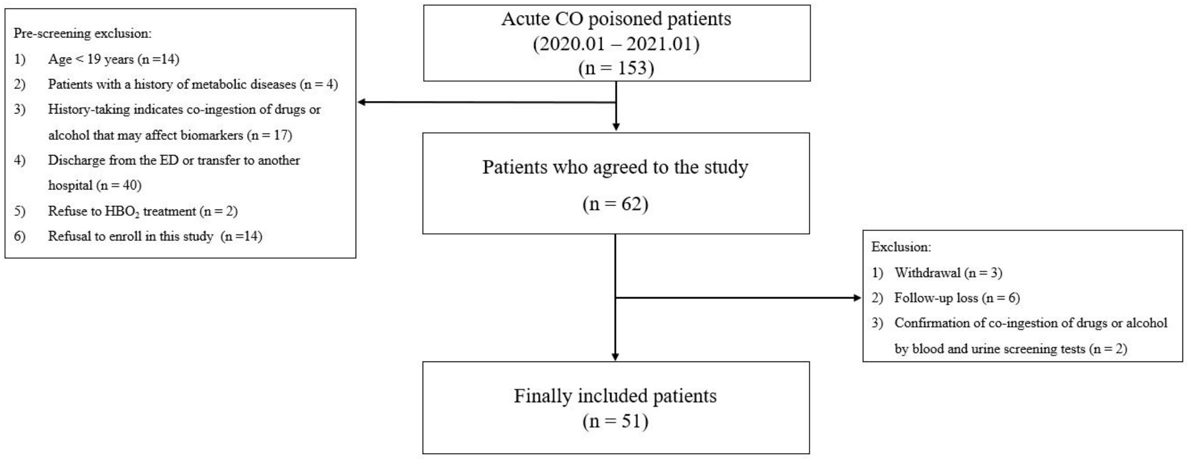

Supplementary Figure S1. Study flow diagram

CO, carbon monoxide; HBO_2, hyperbaric oxygen; ED, emergency department

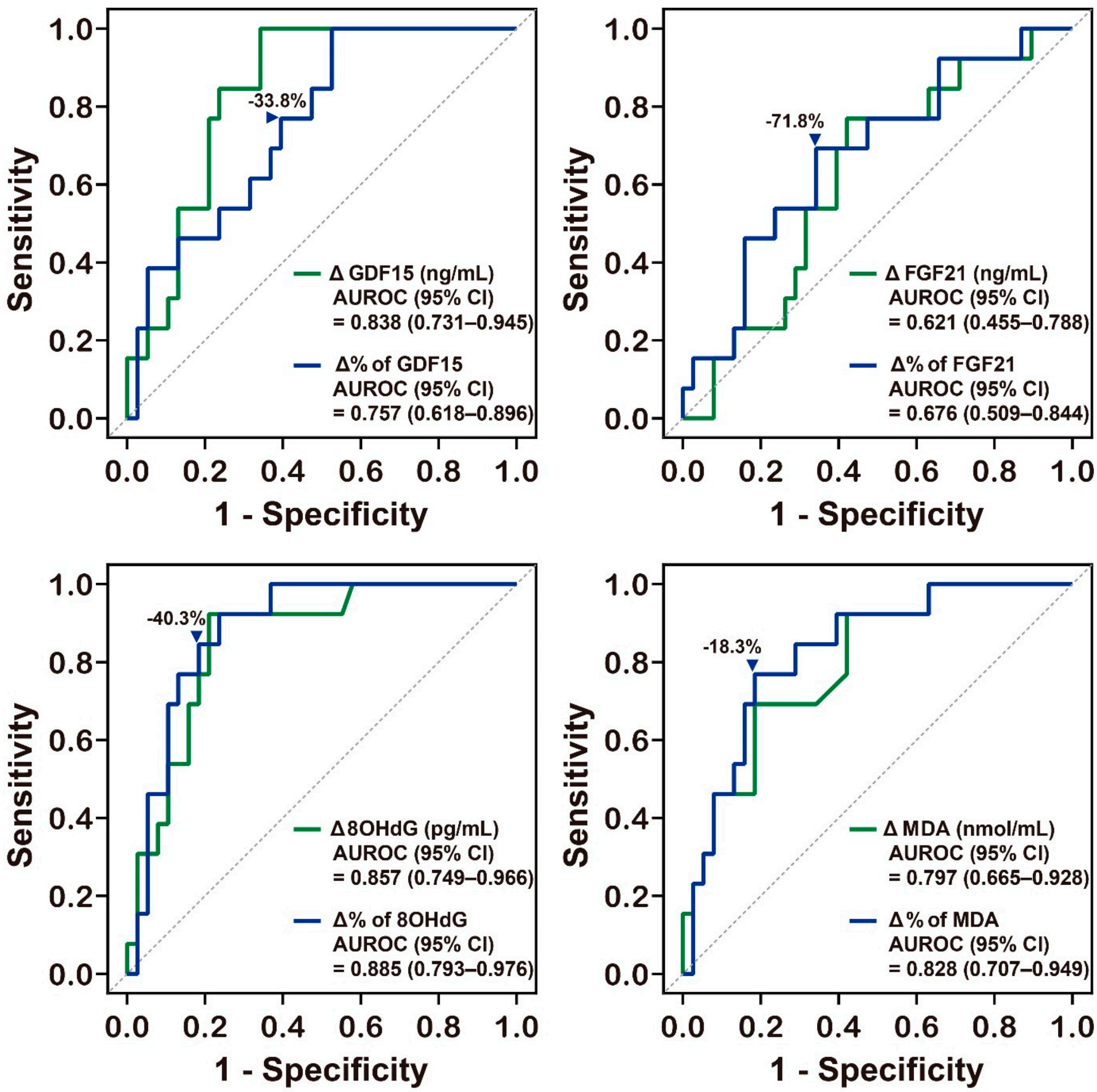

Supplementary Figure S2. Receiver operating characteristic curve analyses of stress biomarkers for predicting neurocognitive outcomes following CO poisoning.

The delta (Δ) means the difference between 1-day post-HBO_2 therapy and baseline values. The delta-percentage (Δ%) was defined as [(1-day post HBO_2 value − baseline value) / baseline value test) × 100]. The optimal cutoff values of Δ% (▼) were defined according to the highest Youden index.

Supplementary Table S1. Global deterioration scale

Stage	Cognitive dysfunction	Clinical characteristics
1	No cognitive decline	*Patients appear clinically normal.*<br><br>No complaints of memory deficits.<br><br>No evident memory deficit on clinical interview.
2	Very mild cognitive decline	*Patients complain of memory deficits.*<br><br>Most frequently, patients:<br><br>(a) forget where they have placed familiar objects<br><br>(b) forget the name of someone they formerly knew well.<br><br>No objective evidence of memory deficit on clinical interview.<br><br>No objective deficits in employment or social situations.<br><br>Patients display appropriate concern about their symptoms.
3	Mild cognitive decline	*Earliest clear-cut deficits.*<br><br>Objective evidence of memory deficit is obtained only through an intensive interview conducted by a trained geriatric psychiatrist. Concentration deficit may be evident on clinical testing.<br><br>Patients may demonstrate a reduced ability to:<br><br>(a) remember names upon introduction to new people<br><br>(b) retain information after reading a passage from a book

		Decreased performance becomes manifest in demanding employment and social situations. Examples may include:<br><br>(a) coworkers becoming aware of the patient's relatively poor performance<br><br>(b) difficulties in finding words and names becoming evident to intimate acquaintances<br><br>(c) losing or misplacing objects of value<br><br>(d) getting lost when traveling to unfamiliar locations<br><br>The subtlety of the clinical symptoms may be exacerbated by the denial that is often manifest in these patients. Mild-to-moderate anxiety also accompanies the symptoms, typically when the patients are forced to cope with challenging employment and social demands that they find they can no longer negotiate.
4	Moderate cognitive decline	*Clear-cut deficits on careful clinical interview.*<br><br>Deficits are manifest in many areas, such as:<br><br>(a) concentration deficit elicited in serial subtractions<br><br>(b) decreased knowledge of current events and recent life events<br><br>(c) upon careful questioning, patients may exhibit a deficit in the memory of their personal history<br><br>(d) decreased ability to travel alone and manage finances<br><br>Patients can no longer perform complex tasks accurately and efficiently. However, certain abilities remain preserved, such as:

		(a) orientation to time and people<br><br>(b) familiar persons and faces can be distinguished from strangers<br><br>(c) ability to travel to familiar locations<br><br>Denial is often the dominant defense mechanism. The evident decline in the patients' intellectual and cognitive capacities is too overwhelming a loss for full conscious acceptance and recognition. A flattening of affect and withdrawal from situations previously considered challenging are observed.
5	Moderately severe cognitive decline	<u>*Patients can no longer survive without some assistance.*</u><br><br>During interviews, patients are unable to recall a major relevant aspect of their current lives. Examples include:<br><br>(a) difficulty recalling their address or telephone number, names of close family members, such as grandchildren, or the name of the high school or university from which they graduated<br><br>(b) some disorientation with time (date, day of the week, season) or location<br><br>(c) well-educated patients may have difficulty counting backwards from 40 by fours or from 20 by twos.<br><br>Patients retain the knowledge of many major facts regarding themselves and others. They invariably know their own names and generally know their spouse's name and their children's names. They require no assistance with toileting and eating, but may have some difficulty choosing the proper clothing to wear and may occasionally clothe themselves improperly (e.g., put their

		shoes on the wrong feet).
6	Severe cognitive decline	*Patients may occasionally forget the name of their spouse, on whom they depend entirely for survival.*<br><br>Patients are largely unaware of all recent events and experiences in their lives.<br><br>They retain some knowledge of their past, but this knowledge is very uncertain. They are generally unaware of their surroundings, the year, or the season and may have difficulty counting backward, and sometimes forward, from 10. Patients require substantial assistance with activities of daily living. These symptoms are quite variable and include:<br><br>(a) delusional behaviour (e.g., patients may accuse their spouse of being an impostor, may talk to imaginary figures in the environment, or to their own reflection in the mirror)<br><br>(b) obsessive symptoms (e.g., continual repetition of simple cleaning activities)<br><br>(c) anxiety symptoms, agitation, and previously nonexistent violent behaviour<br><br>(d) cognitive abulia (i.e., loss of willpower because they cannot carry a thought long enough to determine a purposeful course of action).
7	Very severe cognitive decline	*All verbal abilities are lost.*<br><br>Frequently, there is no speech ability at all; only grunting remains.<br><br>Patients have urinary incontinence and require assistance with toileting and eating. They lose psychomotor skills

(e.g., the ability to walk). The brain appears unable to tell the body what to do. Generalised cortical and focal neurologic signs and symptoms are frequently present.

Supplementary Table S2. Predictive performance of the CO prognosis index for the neurocognitive outcome of post-CO poisoning

	**Optimal cutoff value**	**SN (%)**	**SP (%)**	**LR+**	**LR–**	**PPV (%)**	**NPV (%)**	**Accuracy (%)**
CO Prognosis Index	32	92.3 (64.0–99.8)	92.1 (78.6–98.3)	11.7 (3.9–35.0)	0.08 (0.01–0.55)	80.0 (57.2–92.3)	97.2 (84.2–99.6)	92.2 (81.1–97.8)

SN, sensitivity; SP, specificity; LR+; positive likelihood ratio; LR–, negative likelihood ratio; PPV, positive predictive value; NPV, negative predictive value.
